# “*If it's issues to do with nutrition…I can decide…*”: gendered decision-making in joining community-based child nutrition interventions within rural coastal Kenya

**DOI:** 10.1093/heapol/czx032

**Published:** 2017-12-09

**Authors:** Kelly W Muraya, Caroline Jones, James A Berkley, Sassy Molyneux

**Affiliations:** 1 Health Systems & Research Ethics Department, P.O Box 230-80108, Kilifi, Kenya; 2 Centre for Tropical Medicine & Global Health, Nuffield Department of Medicine, University of Oxford, Old Road Campus, Headington, Oxford OX3 7BN, UK

**Keywords:** Child nutrition, community-based interventions, gender, health systems, rural households

## Abstract

Gender roles and relations play an important role in child health and nutritional status. While there is increasing recognition of the need to incorporate gender analysis in health planning and programme development, there has been relatively little attention paid to the gendered nature of child nutrition interventions. This qualitative study undertaken in rural Coastal Kenya aimed to explore the interaction between household gender relations and a community-based child nutrition programme, with a focus on household decision-making dynamics related to joining the intervention. Fifteen households whose children were enrolled in the programme were followed up over a period of 12 months. Over a total of 60 household visits, group and individual in-depth interviews were conducted with a range of respondents, supplemented by non-participant observations. Data were analysed using a framework analysis approach. Engagement with the intervention was highly gendered with women being the primary decision-makers and engagers. Women were responsible for managing child feeding and minor child illnesses in households. As such, involvement in community-based nutrition interventions and particularly one that targeted a condition perceived as non-serious, fell within women’s domain. Despite this, the nutrition programme of interest could be categorized as gender-blind. Gender was not explicitly considered in the design and implementation of the intervention, and the gender roles and norms in the community with regards to child nutrition were not critically examined or challenged. In fact, the intervention might have inadvertently reinforced existing gender divisions and practices in relation to child nutrition, by (unintentionally) excluding men from the nutrition discussions and activities, and thereby supporting the notion of child feeding and nutrition as “women’s business”. To improve outcomes, community-based nutrition interventions need to understand and take into account gendered household dynamics, and incorporate strategies that promote behaviour change and attitude shifts in relation to gendered norms and child nutrition.

Key Messages

- Engagement with community-based nutrition interventions can be highly gendered with implications for uptake and use of the interventions. It is therefore important to incorporate gender analysis in design and implementation of such interventions.
- Gender-sensitive and particularly gender-transformative approaches should be encouraged for better health outcomes for children and the society at large.

## Introduction

Nutrition impacts significantly on early child development (Black *et al.* 2008, 2013) . Gender and related roles and relations also have an important influence on child health and nutritional status. In 1999, Haddad summarized the significance of women’s status to child survival by presenting a gendered version of the UNICEF Framework of determinants of child undernutrition. At each strata of the framework, he illustrates that any prejudicial practices against women adversely impact on child health and nutritional status (Haddad 1999). For example, differential intra-household allocation of food based on sex difference results in undernourished girls who on attaining reproductive age, tend to bear small-for-gestational-age babies. These babies in turn experience a range of developmental problems (Haddad 1999; Black *et al.* 2013), which propagates the inter-generational effects of undernutrition (Haddad 1999; Madise *et al.* 1999; Food and Agriculture Organization of the United Nations 2000; Welthungerhilfe *et al.* 2010). Likewise, disparity in control of household resources dictated by gender-based norms of resource distribution may inhibit a mother’s ability to access adequate health care or nutritionally suitable foods for her children (Howard 1994; Haddad 1999; Molyneux *et al.* 2002; Abubakar *et al.* 2011). Inadequate political and legal structures that undermine women’s basic rights, that in turn lead to wider gender disparities in income generation and other activities such as educational investment, also unfavourably impact on women’s ability to care and provide for their children (Haddad 1999).

The 2013 *Lancet* series on maternal and child nutrition highlighted the links between maternal status and child health and nutrition including an association between improved women’s status and enhanced child nutrition (Black *et al.* 2013; Ruel *et al.* 2013). For instance, maternal nutritional status at the point of conception and during pregnancy was found to be vital to foetal growth and brain development (Bhutta *et al.* 2013; Black *et al.* 2013;). Maternal iron deficiency was associated with detrimental birth outcomes, including low weight in babies at birth, and increased perinatal mortality (Black *et al.* 2013). Maternal stunting was linked to foetal growth restriction and low weight in children, both of which account for a significant proportion of deaths in children less than 5 years of age (Black *et al.* 2013). Suboptimum breastfeeding and maternal undernutrition were each shown to account for >800 000 deaths annually in children younger than 5 years and neonates (Bhutta *et al.* 2013; Black *et al.* 2013). In addition, maternal depression is a significant determinant in mediating caregiving practices and health seeking behaviour, and was associated with poor child nutrition and development (Ruel *et al.* 2013). Importantly, nutrition-sensitive interventions that specifically target women or incorporate a women’s empowerment component, such as increased control over income from the sale of merchandise, were consistently shown to have a positive effect on child nutrition (Ruel *et al.* 2013).

Initiatives to address child undernutrition focus on either prevention or treatment, with increasing emphasis on community-based approaches in the management of undernutrition (Haines *et al.* 2007; Bhutta *et al.* 2008, 2013; Ministry of Public Health & Sanitation Kenya *et al.* 2009; Lemma and Matji 2013; Ruel *et al.* 2013). There is growing recognition of the need to undertake gender analysis in health planning and programme development—including increasing involvement of men in health interventions—as well as to incorporate evidence-based strategies that enhance gender equity and promote both men and women’s empowerment for improved health outcomes (Vlassoff and Moreno, 2002; Kim *et al.* 2007; Rath *et al.* 2010; Muralidharan *et al.* 2014). Health programmes can broadly be categorized into those that are gender-blind and those that are gender-aware or gender-sensitive (Muralidharan *et al.* 2014). In a recent systematic review of gender-integrated health programmes in low and middle income countries, gender-blind programmes were described as those “*…that do not demonstrate awareness of the set of roles, rights, responsibilities, and power relations associated with being male or female*“ (p.16) (Muralidharan *et al.* 2014). Gender-aware programmes on the other hand recognize and give due consideration to the influence of gender roles and norms on various aspects of health, including use and access of services, as well as eventual health outcomes (Muralidharan *et al.* 2014). Such interventions deliberately incorporate gender as a core part of their design, implementation and monitoring and evaluation; and can be categorized along a continuum dependant on their particular approach to gender-integration. Broadly, however, these latter interventions can be grouped as those that are “gender-accommodating” and those that are “gender-transformative” (Muralidharan *et al.* 2014).

Gender-accommodating programmes take into account existing gender dynamics, practices and inequalities, and design and implement the intervention around these barriers (Muralidharan *et al.* 2014). Thus, gender-accommodating programmes adapt and adjust for inequitable gender roles and relationships, and engage communities to spread information and support behaviour change (Muralidharan *et al.* 2014). Despite potentially achieving positive gender-related outcomes, gender-accommodating programmes do not necessarily challenge existing inequalities, promote critical reflection on gender norms and practices or put in place strategies that can result in fundamental shifts in attitudes that can raise the status of women (and men).

Gender transformative programmes on the other hand go beyond adjusting for existing gender norms and inequalities and critically examine, challenge and address these issues. These interventions use culturally appropriate strategies to contest rather than adapt to the status quo with regards to gender inequalities and power imbalances. The ultimate aim of such interventions is to change attitudes, foster behaviour change and empower women, men, boys, girls and other sexual minorities (Muralidharan *et al.* 2014). Gender transformative interventions generally draw on participatory approaches that encompass awareness-raising, increased community participation and empowerment activities that contribute to a shift in attitudes and practices, which ultimately lead to improved health outcomes for the communities involved. Literature from low- and middle-income countries shows that gender-aware programmes are generally noted as enhancing health knowledge, attitudes and behaviour, as well as improving health outcomes (Muralidharan *et al.* 2014). In addition to that, gender-transformative programmes influence gender-equitable attitudes, raise women’s self-confidence and self-efficacy, and contribute to increased joint decision-making by men and women (Muralidharan *et al.* 2014).

Studies examining the gender orientation of health interventions have primarily focused on areas such as sexual and reproductive health, family planning and HIV programmes (Varkey *et al.* 2004; Muralidharan *et al.* 2014). There has, however, been less attention on the gendered nature of child nutrition programmes, and the role of gender in decisions around the management of child undernutrition. The limited available literature suggests that men have largely been overlooked in child nutrition efforts, with interventions focusing mainly on women and other female stakeholders such as grandmothers, and have advocated for more gender accommodating programmes to improve effectiveness (Thuita 2011; Kuyper and Dewey 2012). Existing literature is, however, silent on the use of gender transformative approaches in child nutrition to challenge-related gender-inequitable norms and practices and encourage shifts in thinking and attitudes. The work presented in this article was part of a broader 4-year study exploring the interaction between an existing community-based child nutrition programme and household gender relations. This article focuses specifically on decision-making related to joining the programme, and suggests ways in which such programmes can incorporate strategies to be more gender transformative.

## Methods

### Study site

This qualitative study was undertaken in Kilifi County, coastal Kenya, focusing on one rural sub-location within the County. The majority of residents are Giriama with family structures being predominantly extended, particularly in the more rural areas, and polygamy being widely practised. The primary economic activity is rain-fed small scale subsistence farming. The county has low literacy levels, especially amongst females, very high levels of poverty, and frequent food insecurity (Chuma *et al.* 2006; Marsh *et al.* 2008, Muinde, 2011). In addition, Kilifi has high rates of child undernutrition. A survey undertaken in the area in 2011 placed overall acute malnutrition at 4.0% and severe acute malnutrition at 0.7% (Muinde 2011). The prevalence of stunting was extremely high at 48.8 and 19.6% for severe stunting, while overall underweight was 21.3% with severe underweight at 5% (Muinde 2011). As such, food programmes either targeting food insecure households or undernourished children have existed in the study area for several years, with multiple programmes often occurring concurrently (Muraya 2014). For instance, there were five different food programmes happening simultaneously in the study sub-location at the time of this study. Within the study community, child nutrition and care including management of minor child illnesses is primarily the responsibility of women (Molyneux *et al.* 2002; Muraya, 2014), with men mostly regarded as food or financial providers (Muraya 2014). Women are also the ones who predominantly access health facilities in relation to child health - whether for routine child growth monitoring or to seek treatment for minor ailments—and have been observed to have some level of autonomy in treatment decision-making for illness considered as non-severe (Muraya *et al.* 2016).

### Nutrition programme of interest

The focus of this study was the household (and therefore uncomplicated malnutrition managed at the household level), and on programmes targeting children beyond the recommended exclusive breastfeeding phase. On the basis of a situational analysis of all nutrition interventions in the area, the Targeted Supplementary Feeding Programme (SFP)—a community-based nutrition intervention targeting children aged 6–59 months with moderate acute malnutrition and no clinical symptoms—was identified as a case intervention. The primary objective of the SFP is to improve the nutritional status of participating children, preventing further decline into severe undernutrition that requires hospitalization. The intervention is delivered through health facilities, and entails distribution of food supplements in the form of fortified corn-soy blend flour mixed with vegetable oil, or a ready-to-use supplementary food known as Plumpy-Sup^®^. Carers of enrolled children (usually mothers and grandmothers) are expected to collect the intervention product every fortnight from the participating health facility where they are registered, and utilize it at home according to given instructions. The intervention is intended to be a holistic approach with a sustainable impact involving in addition to the food supplementation, nutritional counselling to encourage behaviour change, follow-up home visits by community health workers and management of common ailments at the health facility. The SFP is part of a larger scale initiative known as the Community-based Management of Acute Malnutrition (CMAM) programme. CMAM was established in mid-2009 under a tripartite agreement between the Kenyan Ministry of Health, World Food Programme and UNICEF, and is ongoing. Implementation of the CMAM Programme is largely guided by the National Guidelines on CMAM (Ministry of Public Health & Sanitation Kenya *et al.* 2009) which act as a roadmap for delivery of the intervention.

### Selection of study households and data collection

Using a selected local health facility (dispensary) as an entry point, 15 households with 24 children engaged in the SFP were each visited three to six times over a 12-month period, totalling 60 visits. The particular dispensary was selected on the basis of having the most active nutrition programme within the study locality, with a total of 95 participants. Following verbal consent, a short descriptive questionnaire was administered to primary carers of all the enrolled children to collect basic information about their households. Using these data, the 15 households were then purposively selected to reflect the diversity of homes involved in the programme. In particular, household structure and headship are central to family dynamics and health decision-making (Whyte and Kariuki 1991; Cosminsky *et al.* 1993; Howard 1994; Molyneux *et al.* 2002; Aubel *et al.* 2004; Bezner Kerr *et al.* 2007; Bezner Kerr *et al.* 2008; Mwangome *et al.* 2010), and were key selection criteria. Other considerations in selecting households for the longitudinal study included level of engagement with the SFP (prolonged engagement vs newly recruited), primary carer’s education level, and husband’s residency where applicable.

**Figure 1.**
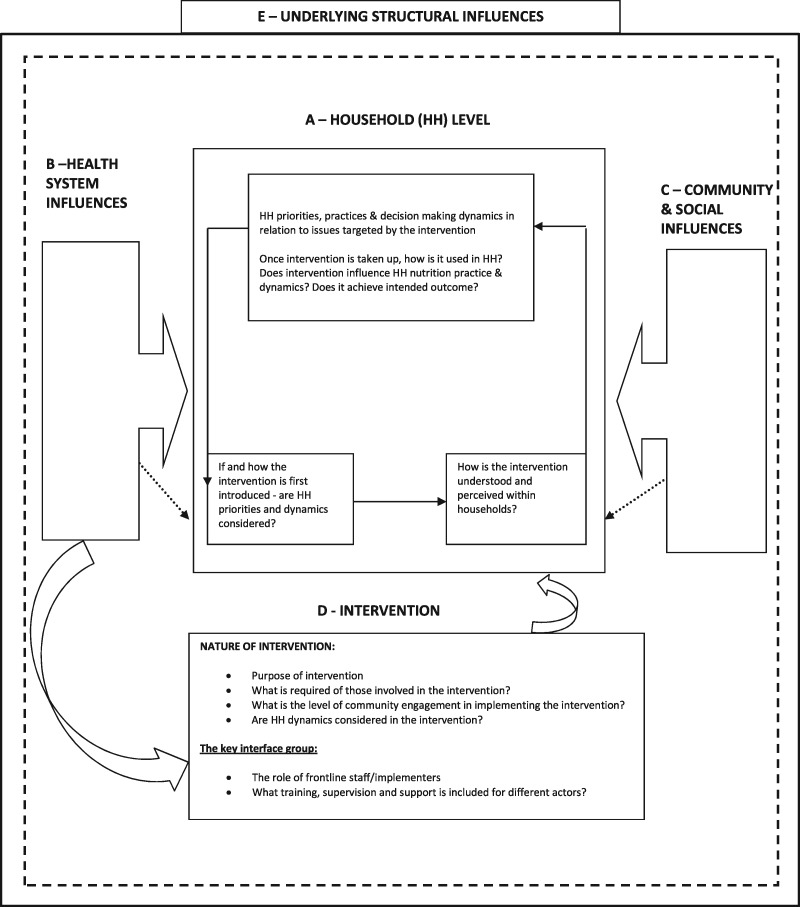
Factors influencing intervention use & uptake

Individual and group in-depth interviews were conducted with a range of respondents within the households. Respondents typically included parents or primary carers of the target children; grandparents, aunts and uncles of the target children; and co-wives of the target children’s mothers in the case of polygamous households. Using open-ended interview guides, a range of topics relating to child and household feeding practices, child health and treatment actions during child ill health, and experiences with community-based nutrition interventions particularly the SFP, were covered during the household visits. The interview guides were developed by the primary author in consultation with the entire research team and were based on the conceptual framework for this study (Figure 1), the study aims and objectives, and factors identified from literature as being important to child health and nutrition and related decision-making. Interviews were audio-recorded and conducted in the local Giriama language, and later transcribed and translated into English. Interviews were complemented with non-participant observations (using an observation checklist) within the households and in selected health facilities that were implementing the SFP. Two field staff—one male and one female—who themselves came from the local community were the primary data collectors, and also provided valuable contextual information. In addition to accompanying the field staff to all household visits, the primary author had regular and continued engagement with both the funders and frontline implementers of the SFP.

### Data analysis

The data from this study were managed using Nvivo 10 and analysed using a framework analysis approach (Green and Thorogood 2009). After immersion in the data, an initial content analysis was undertaken using pre-determined and emergent recurrent themes that together formed the coding scheme. The coding process involved splitting and rearranging the data from all the households and respondents according to thematic content. Coding charts were then developed to enable comparisons between and within households, as well as between the different types of respondents. Finally, relationships and associations between concepts were explored, as well as linking the findings to wider literature and theory. The entire research team was involved in the data analysis process which allowed for extensive consultation and discussion on emerging issues and development of the coding scheme and charts. Furthermore, given the iterative nature of the study, preliminary findings were discussed with participants in later household visits, which enabled discussion of the researchers’ interpretation of the findings. In this way, the respondents became a part of the data analysis process and contributed to further enrichment of the data. Ethical approval was obtained from the authors’ institute.

Written informed consent was obtained from all participants of the longitudinal household study. For participant anonymity and confidentiality, all data have been de-identified and family names have been replaced with pseudonyms.

## Results

### Summary of households under study

The household was defined as a group of people residing collectively in the same compound and answerable to the same overall household head (Chuma *et al.* 2002; Chuma 2005). Of the 15 households, 7 were polygamous while 8 were non-polygamous. Within both categories there were male- and female-headed households. Household size varied from smaller nuclear families consisting of parents and their children to much larger extended families comprising of several semi-autonomous households living within one compound. The households were highly diverse with complex dynamics. Table 1 gives a summary of the various household (HH) structures, the number of visits per household and the types of respondents interviewed.

**Table 1.** Summary of family structures and types of respondents

**HH**^a^**Code**	Type of household					No. of HH visits	Respondents interviewed
	Male	Female	Polygamous	Nuclear	Extended		
Chome	✓		✓		✓	4	Mothers to the target^b^ children (co-wives)
Furaha	✓		✓		✓	6	Parents to the target child Grandparents to the target child
Kazungu	✓		✓		✓	4	Grandmothers to the target children (co-wives)
Katana	✓		✓		✓	4	Parents to the target child Mother’s co-wife
Kenga	✓		✓	✓		3	Mother to the target child Mother’s co-wife
Kadzo		✓	✓		✓	6	Mothers to the target children Father to one of the target children Grandmothers to the target children Aunts to the target children
Zainabu		✓	✓	✓		3	Mother to the target child Mother’s co-wife
Karisa	✓				✓	5	Parents to the target children Grandparents to the target children
Safari	✓				✓	4	Mothers to the target children Father to one of the target children Uncle to the target children
Jasho		✓			✓	3	Parents to the target child
Pendo		✓			✓	4	Mother to the target child Grandmother to the target child Aunts & uncles to the target child
Bakari	✓			✓		4	Mother to the target child
Hamisi	✓			✓		3	Parents to the target child
Mapenzi		✓		✓		4	Mother to the target child
Amina		✓		✓		3	Mother to the target child

a HH refers to household

b Refers to the child(ren) enrolled in the nutrition programme of interest

### Gendered decision-making—joining the SFP

Gender emerged as a key feature in the management of child nutrition in the study households. The role of gender in perceptions of the cause and management of illnesses related to child undernutrition has been reported elsewhere (Muraya *et al.* 2016). Gender also played an important role in engagement with the SFP, with women being the primary decision-makers and engagers with the programme throughout the entire period when children were enrolled. During our 12-month interaction with the study households as well as observations at the selected health facilities, women were noted as the key participators of the programme. This was both with regards to collecting the supplemental food for the targeted children from the health facilities, and maintaining continued engagement with the programme. This article specifically focuses on the role of gender in decision-making related to joining the intervention.

Despite the diversity, in all of the households the main female carer—usually the targeted child’s mother and sometimes the grandmother—made an autonomous decision about joining the programme once they were informed by the health workers of their child’s eligibility. The willingness and ability of mothers to make an independent decision regarding enrolment of their children into the programme was not entirely surprising. The SFP targeted moderate acute malnutrition, a condition which was considered to be non-serious in this community, and therefore within the realm of women’s everyday responsibility of caring for their children (Muraya *et al.* 2016). In addition, there was a tendency to normalize involvement of children in such programmes given the long history of nutrition interventions in the study area (Muraya *et al.* 2016). Thus, enrolment of children into the SFP was not necessarily considered as treatment-seeking for ill health requiring consultations with other family members, but rather viewed as part of routine child care which fell firmly within women’s domain. For instance, this is what one mother stated with regards to her child joining the programme:

*“If it’s a difficult issue concerning the child, we inform her father…like if the child is seriously sick [but] if it's issues to do with child nutrition, my co-wife and I can decide and that’s alright.”* (Katana household, target child’s mother)

This was in contrast to for example, members of the same (Katana) household discussing enrolment of the same child into an ongoing malaria vaccine trial that was occurring in the community at the time of this study. The vaccine trial entailed repeated drawing of blood from participating children—an action that was perceived to have potentially severe adverse consequences, especially in the context of sensitivities around blood within this particular community (Molyneux *et al.* 2004, 2005). In this case, women could not independently decide to enrol their children in the vaccine study without the consent of the male household head:

*“When the young man [vaccine trial fieldworker] came, he spoke to me first because I am the head of the home. They have to give me the explanations first. You can’t just join and you know your child's blood will be taken. As the man, I made the decision but I also consulted with the mother of the child”* (Katana household, male household head)

Specifically with regards to the SFP, discussions with other household members about joining were generally only held when there was a gap between the carer being notified of their child’s eligibility for the programme and the actual enrolment. Carers were usually informed of their children’s eligibility when they visited the health facility for routine child growth monitoring or to seek medical care for minor ailments. When these visits coincided with a designated “SFP product distribution day”, children could be enrolled immediately. If not, carers had to return at a later date to formally enrol their children into the programme. Even when enrolment had been delayed, all of the participating women indicated that if informed of their children’s eligibility on a day when the programme was happening they would immediately enrol their children without the need for consultation and then later explain to other family members.

*“I didn’t take the [SFP] flour on that day when they told me about it. I came home and informed my co-wife then when the [designated] day came, I went and collected the flour. I informed my co-wife because she is the one I am answerable to, she is more senior…But if I had gone on a flour distribution day, I would have collected the flour and then come and explain to her.”* (Kenga household, target child’s mother)

### Other gendered influences—role of other actors in joining the SFP

Despite clear perceptions of autonomy in decisions to enrol their children in the SFP, female carers often received advice and input from family and community members, all of whom had an important influence in the decision-making process. Community members—especially mothers within the community—had significant influence on fellow mothers with regards to joining the programme. This was either through passing on information about the SFP and advising other mothers whose children were perceived as being underweight or unhealthy, or as a result of their being involved in the programme and subsequent positive reports which in turn boosted mothers’ trust in the programme. In nearly all of the households, the targeted children’s mothers mentioned fellow mothers, friends or neighbours as having somehow influenced their decision to enrol their children in the intervention.

*“I went to my neighbour…and explained to her my child’s condition and she told me her children were the same way. They had low weight so she went and talked with the doctors at the Dispensary, and they told her to return on a certain date, so that her child can be enrolled in the programme…I went to her because she had a child like mine and she was already involved in the programme. So I wanted her to give me more information about it…”* (Mapenzi household, target child’s mother)

Family members, particularly grandmothers, also played a role in children being enrolled in the SFP. In some households for example, grandmothers who were concerned about the apparent poor health of their grandchildren encouraged the mothers to seek help from the health workers.

*“The child’s grandmother also told us, “these children who you go with to the clinic all the time and their weight is always low, why don’t you go and talk to those ones who are distributing the [SFP] flour?”…She also advised us to go and talk to the other mothers whose children were already involved in the programme.”* (Kadzo household, target child’s mother).

Men were largely absent with regards to the SFP, and indeed comprised a smaller number of the respondents for this study albeit unintended. The majority of the men were often not present when we visited the households either because they lived and worked elsewhere and only occasionally visited the family home, or were engaged in various daily activities outside the home. Furthermore, even when the men were present during the household visits, they were generally not interested in engaging in the discussions as they described child nutrition as “women’s business”. Our observations during the 12 months of interacting with the study households, as well as observations at selected health facilities during the “SFP distribution days”, support this notion of child nutrition as being primarily “women’s zone”. The view of moderate malnutrition as being non-serious, the normalcy associated with involvement in nutrition programmes and the subsequent autonomy of women in related decision-making could also explain this general absence of men.

## Discussion & recommendations

Fifteen households were followed up over a period of 12 months to explore the role of gender in decision-making related to joining a community-based child nutrition programme. Engagement with the SFP was highly gendered with women, in their role as carers, being particularly exposed to the programme. Mothers, and occasionally grandmothers, were responsible for taking children to the health facility where information about the programme was provided and their children’s eligibility assessed. Discussions about the programme occurred primarily within a female domain with concerned mothers actively seeking out health workers and other (female) community members in relation to their children’s health and nutritional status. Furthermore, decision-making power existed primarily within the female sphere. Mothers and/or senior women were the key decision-makers in relation to joining the SFP and eventual engagement with the programme. Men hardly featured in discussions or decisions about enrolment or use of the intervention within the home. The absence of men’s voices in programme uptake and use is perhaps not surprising given that in this community, child nutrition and the management of perceived minor child illnesses fell squarely within women’s domain (Muraya *et al.* 2016). As such, enrolment in a nutrition programme targeting moderately undernourished children—a condition that was perceived as non-serious—was within women’s normal areas of operation, and would explain women’s agency in the related decision-making.

The role of gender in treatment seeking for child illness is widely recognized (Castle 1993, Agarwal 1997; Haddad 1999; Marinda 2006, Hampshire *et al.* 2009). Studies have shown that women are often held ultimately responsible for the health of their children, but many household members and other social network members can be involved in treatment-seeking actions (Whyte and Kariuki 1991; Cosminsky *et al.* 1993; Feyisetan *et al.* 1997; Amuyunzu 1998; Bezner Kerr *et al.* 2008; Tolhurst *et al.* 2008; Richards *et al.* 2013). Thus, whether or not mothers make independent decisions regarding the treatment of child illnesses is determined by a range of inter-related factors including the nature and perceived severity of illness, perceived child ownership, perceived cause of illness and intra-household roles and relations (Molyneux *et al.* 2002; Tolhurst and Nyonator 2006; Tolhurst *et al.* 2008). The findings of this study have some similarities with work conducted in the same study area by Molyneux *et al.* (2002) exploring decision-making dynamics and treatment-seeking patterns for fever and malaria. Molyneux *et al.* (2002) found that while in general, men and older women were the main decision makers for treatment of child illness, women were less likely to consult, or seek assistance or permission from anyone for symptoms such as fever that were considered to be normal or every day. Work undertaken in rural Ghana by Tolhurst and colleagues on treatment seeking for child illness and related decision-making showed similar dynamics (Tolhurst and Nyonator 2006; Tolhurst *et al.* 2008). This situation was seen across these studies to change for more serious illness because of greater potential implications for the child’s health and for household costs. The latter related to treatment-seeking (travel, admission and treatment costs), and to loss of income as a result of caring for sick children. Therefore, in both the Kilifi and Ghana studies, women had more control over less serious childhood illness and less autonomy over child health conditions perceived as serious or complex.

Despite the crucial role of gender in child nutrition and management of childhood illness both within the study setting and more broadly, and the evident gendered nature of the SFP, the programme can be categorized as being gender-blind. Gender was not explicitly considered in the design and implementation of the programme, and gender roles and norms in the community with regards to child nutrition were not critically examined or challenged. There were also no strategies incorporated into the programme to adapt and adjust for existing gender norms, promote behaviour change and attitude shifts in relation to gender and child nutrition, or that would empower or raise the status of men and women. Discussions with funders and implementers of the intervention as well as review of relevant documents confirmed this. In fact, the programme might have inadvertently reinforced existing gender divisions and practices in relation to child nutrition, underpinning the notion of child feeding and nutrition as “women’s business”. Furthermore, the National Guidelines on CMAM (Ministry of Public Health & Sanitation Kenya *et al.* 2009) which guide delivery of the SFP place particular emphasis on the role played by household members in preventive and promotive nutrition care, as well as in managing clinical care of affected individuals. Despite this, and the potential gendered household roles and responsibilities in relation to health and nutrition, the guidelines only refer to gender once and specifically in the context of an emergency nutrition response. In this particular context, the guidelines state that in identifying the main health and nutrition problems during a (humanitarian) crisis, “*…data collected should be disaggregated by age, gender…*” (p. 152). Gender and related norms, roles and responsibilities do not however feature in any other component of the guidelines.

The importance of integrating gender into health programmes is widely acknowledged (; Vlassoff and Moreno 2002; Varkey *et al.* 2004; Kim *et al.* 2007; Rath *et al.* 2010; Muralidharan *et al.* 2014). In particular, literature suggests that transformative approaches that challenge and promote critical reflection on existing norms and inequalities, include participatory methods that engage the wider community and include empowerment strategies, should be encouraged. As has been observed in other parts of Kenya (Thuita 2011), this study shows that in this particular rural community, child nutrition is considered to be primarily the responsibility of women. As such, programmes such as the SFP that do not take into account existing community norms, views and practices have the potential to further underpin these notions. On the other hand, allowing for such considerations might result in differently designed or structured community-based nutrition interventions that for example, actively and meaningfully engage men and other community stakeholders to shift thinking around child nutrition. This would necessitate innovative approaches that result in more gender-equitable attitudes that frame child nutrition as a “concern for all”.

In particular, embedding culturally sensitive participatory approaches within nutrition interventions that encompass awareness-raising including targeting existing men and women’s community groups or use of theatre and media, drawing on available resources such as the use of well-respected “community champions” in both male and female spheres, increased community participation of both men and women in nutrition-related activities and campaigns and empowerment activities such as microfinance and income generating activities for vulnerable groups, could be useful. Such strategies could plausibly result in a re-thinking and prioritization of child nutrition within households and communities, and a better understanding of the potential adverse consequences of poor child nutrition including increased vulnerability to illness, longer-term developmental and economic problems of stunting including loss of productivity and the inter-generational effects of stunting. This could ultimately result in better health outcomes for women, children and the community at large. Furthermore, to be inclusive, community-based nutrition programmes need to be delivered in locations that are convenient and easily accessible to both men and women. Literature shows that men are less likely than women to access primary health care facilities (Hawkes 1998; Hartigan 2001). Thus, programmes such as the SFP that are delivered through such facilities may unintentionally exclude men from the process.

In addition, as suggested in literature, nutrition-sensitive transformative approaches such as women’s economic empowerment, increased educational opportunities for girls and women, and strengthening communication and negotiation skills could also contribute to increased joint decision-making power for men and women, and possibly (though not automatically) greater financial independence and self-confidence for women (Bhutta *et al.* 2013; Ruel *et al.* 2013; Muralidharan *et al.* 2014). This in turn could potentially have positive implications for child nutrition such as mothers’ increased ability to purchase nutritious foods for their children and determine their children’s food choices, as well as better access to health services when required and overall improved gender-equitable attitudes.

### Limitations

The household was the focus of this study and thus the findings are primarily from the perspective of the intervention users. While this is important and provides a rich account of experiences from those who are targeted by the interventions, it is equally important to gain the perspective of the intervention implementers. Interviewing the implementers would have further strengthened this study and given a more holistic understanding of the programme and related issues, and should be considered for future work.

## Conclusion

This study focused on the role of gender in decision-making related to joining a community-based nutrition programme. Women were noted as having significant autonomy in enrolling their children and were the primary engagers with the programme, with men being largely absent. Despite this gendered engagement and existing gender norms and practices in relation to child nutrition within this community, the programme did not explicitly consider gender in either its design or implementation. Consequently, the programme potentially further reinforced the notion of child nutrition as a predominantly “women’s issue”, inadvertently excluding men in the process. Improving child health outcomes and nutritional status requires collective effort involving all relevant members of the family and community. Therefore, nutrition programmes particularly those that are implemented at community or household level, need to be cognizant of existing gendered divisions of roles and responsibilities, and consequently attempt to address or challenge these norms for improved child health outcomes.
